# Cough-Induced Detrusor Overactivity—Outcome after Conservative and Surgical Treatment

**DOI:** 10.3390/jcm13206109

**Published:** 2024-10-14

**Authors:** Anna-Sophie Villiger, Mihaela Madalina Fluri, Diana Hoehn, Anda Radan, Annette Kuhn

**Affiliations:** Department of Obstetrics and Gynecology, Bern University Hospital, University of Bern, 3010 Bern, Switzerland; mihaela-madalina.fluri@insel.ch (M.M.F.); diana.hoehn@insel.ch (D.H.); anda.radan@insel.ch (A.R.); annette.kuhn@insel.ch (A.K.)

**Keywords:** anticholinergic/betamimetic treatment, behavioral therapy, bulking agents, cough-induced detrusor overactivity, mixed urinary incontinence, pad test, patient rating, pelvic floor muscle exercises, midurethral sling

## Abstract

**Background/Objectives**: The most common variant of mixed urinary incontinence is stress-induced urge urinary incontinence with the correlating urodynamic findings of cough-induced detrusor overactivity (CIDO). This prospective study assessed the clinical outcomes and leakage improvement among patients with CIDO following conservative or surgical treatment. **Methods**: We included patients with CIDO treated at our tertiary referral center from January 2018 to July 2021 in this prospective cohort study. The detection of a detrusor contraction after a cough was diagnosed as CIDO by urodynamic multichannel testing. All the patients in our study received personalized care, with behavioral therapy and anticholinergic/betamimetic treatment as a first step. If leakage persisted, patients were given a choice between pelvic floor muscle exercises (PFMEs), periurethral bulking or a midurethral sling. The primary outcome was the mean difference in urine leakage in the pad test before and six months after treatment. **Results**: Thirty-five patients met the inclusion criteria for CIDO and all presented a positive pad test at baseline (mean: 27 g). All 35 patients participated in behavioral therapy and anticholinergic/betamimetic treatment. Twenty-two patients (62.9%) underwent PFME, twelve patients (34.2%) received periurethral bulking, and nine patients (25.7%) received a midurethral sling. After all the treatments, our cohort showed a significant improvement in the pad test (mean: 5.7 g, *p* < 0.001). The result was more favorable after periurethral bulking than the midurethral sling (*p* < 0.001). **Conclusions**: This study shows the effectiveness of conservative treatment as a first step. In cases needing further treatment, bulking agents may be superior to PFME and midurethral propylene slings, offering new perspectives in the field of urogynecology and urinary incontinence.

## 1. Introduction

Up to 50% of women experience UI during their lifetime and 50% of these cases are due to mixed urinary incontinence (MUI), the co-occurrence of stress urinary incontinence (SUI), and urge urinary incontinence (UUI) [1,2,3]. MUI is not only the most common form of urinary incontinence, it is also clinically important. Patients with MUI have more severe incontinence than those with SUI or UUI alone [4]. The International Continence Society (ICS) defines MUI as complaints of both stress and urgency urinary incontinence, i.e., involuntary loss of urine associated with urgency and also with effort or physical exertion, including sporting activities, or on sneezing or coughing [5]. The most common variant of MUI is stress-induced UUI (29–39%), in which UI with urgency immediately follows a physical stress [2]. Physical stress provokes episodes of UUI by means of mechanical and neuromuscular mechanisms [6]. The correlated urodynamic findings are cough-induced detrusor overactivity (CIDO) with or without urinary leakage. Although CIDO is mainly found in patients with MUI, it is clinically classified as SUI because of a positive cough test.

The paucity of data on the treatment and outcomes in CIDO represents a significant challenge to the effective treatment of this condition. The first line of treatment for MUI is to address the predominant complaint [7,8]. When conservative treatments, including behavioral therapy, anticholinergic/betamimetic treatment and pelvic floor muscle exercise (PFME), are not effective enough to reduce the incontinence episodes, then surgery for SUI is prioritized. Bulking agents and midurethral slings are treatment options for SUI. They have high success rates and low complications [9,10,11,12]. Midurethral retropubic slings have an 89% cure rate and a 97% improvement rate at the one-year follow-up [9]. Cure rates of 90% are published 17 years after a retropubic sling [10]. In the initial scientific studies on retropubic midurethral slings, it was observed that urinary urgency frequently co-occurred in women with SUI. Furthermore, the midurethral sling was demonstrated to be an effective treatment for both conditions in a significant proportion of cases [13]. Petros and Ulmsten [14,15] have demonstrated that bladder instability in women may be attributed to premature activation of the micturition reflex that is triggered by vaginal wall laxity [16]. Bulking therapy is an effective and valuable alternative to midurethral slings for patients with MUI [12]. In contrast, the literature on bulking agents is sparse. Hoe et al. recently found short-term success rates of 30–90% and long-term efficacy of 21–80% [11]. We therefore assessed the clinical outcomes, including the leakage improvement, among patients with clinical SUI and urodynamic CIDO after conservative or surgical treatment.

## 2. Materials and Methods

This prospective, observational, single-center study was a collaborative effort conducted at the Division of Urogynecology at the Department of Obstetrics and Gynecology of the Bern University Hospital (Switzerland), a tertiary referral center, between January 2018 and July 2021. The recruitment and exposure period started in January 2018 and ended in January 2021, the follow-up period finished in July 2021, and the data collection terminated in February 2022.

We invited eligible patients with CIDO treated at the Department of Urogynecology to participate. The detection of a phasic contraction of the detrusor < 5 s after a cough with or without leakage diagnosed CIDO by multichannel urodynamics (SEDIA^®^ Giviez, Switzerland) using a standardized protocol published by the Good Urodynamic Practices Guidelines of the International Continence Society [17,18]. Information about the study and instructions about the clinical examination and interventions were given verbally and on a detailed written consent form. Patients provided written informed consent after thorough counseling before any study intervention. We respected the privacy rights of the human participants.

We excluded patients with pure UUI, previous incontinence surgery, pelvic malignancies, radiation therapy of the pelvis, recurrent urinary tract infections, history of psychiatric or neurologic disease, and concomitant pelvic organ prolapse >I° stage according to the Pelvic Organ Prolapse Quantification system [19].

The primary outcome was the mean difference in the pad test urine leakage before and six months after treatment (in grams), defined as >2 g in 1 h after moderate physical activity according to the ICS protocol. The pad test quantifies the amount of urine lost throughout testing, measuring the increase in the pad’s weight (pre- and post-testing) [5]. It measures the severity of incontinence and is perhaps the most objective measure. The severity of incontinence (quantified by the pad weight) affects the outcomes of surgery. The 1 h pad test was used because it is quick and standardized. The secondary outcome was defined as cured, improved, without needing further therapy, improved, needing further therapy, same, or worse than before as determined by the patients’ rating.

All the patients received behavioral therapy and anticholinergic/betamimetic treatment as a first step. If leakage persisted, PFME, periurethral bulking with polyacrylamide hydrogel (Bulkamid^®^) or a midurethral polypropylene sling (TVT exact^®^) were proposed and patients were free to choose their preferred method. Follow-up was performed six months after the therapy.

We performed the statistical analysis using Stata 16 (Stata Corporation, College Station, TX, USA). We calculated the median, range, mean, and standard deviation for continuous variables and the percentages for the qualitative variables. A quantile–quantile plot was employed to ascertain whether the data exhibited a normal distribution. We used Student’s paired *t*-test, linear, and logistic regression for the average comparison. We omitted cases with missing values. A significant correlation was defined as a *p*-value < 0.05.

No approval by the Ethics Committee was required since this was a quality control single-center study describing our standard care according to current Swiss law. Before the examination and therapy, all the patients gave informed consent for using their clinical data in research studies, ensuring data confidentiality. We report using the Strengthening the Reporting of Observational Studies in Epidemiology (STROBE) statement [20].

## 3. Results

Thirty-five patients met the inclusion criteria for CIDO and agreed to participate in this study. No participants dropped out of the study; compliance for completing the follow-up was 100%.

The patients’ demographics, urodynamic characteristics, and therapies are summarized in Table 1. All the patients presented a positive pad test at baseline (median: 23 g). The median bladder volume when CIDO occurred was 189 mL, and 32 patients (91.43%) had a leakage with CIDO. After treatment, the median leakage measured by the pad test was 2 g. All the patients participated in behavioral therapy and anticholinergic/betamimetic treatment. Afterward, seven patients (20%) did not require further treatment, twenty-two patients (62.9%) underwent PFME, and six patients (17.14%) experienced periurethral bulking. After secondary therapy, another six patients (17.14%) underwent periurethral bulking, and nine (25.71%) received a midurethral sling. A total of 21 patients (60%) required surgery, 12 (34.2%) received bulking, and 9 (25.71%) received a midurethral sling. Of all the patients, 14 (40%) were cured or did not require further treatment after conservative therapy.

**Figure 1:**
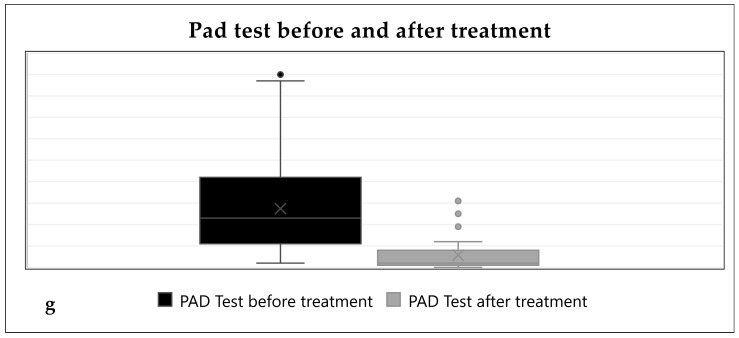
Pad test before and after treatment.

Statistics concerning the preoperative and postoperative pad tests are presented in Table 2. After all the treatments, the cohort showed a significant improvement in the pad test (mean: 5.71 g, *p* < 0.001, 95% CI 14.61–28.77) (Figure 1). Subgroup analysis of the pad test also showed a significant improvement after behavioral therapy and anticholinergic/betamimetic treatment (mean: 2.57 g, *p* < 0.001, 95% CI 14.18–45.53), as well as improvements after PFME, periurethral bulking and midurethral slings. There was no correlation between the primary outcome and the BMI or parity.

We also compared the patients’ ratings to the preoperative and postoperative pad tests (Table 2). Patients with subjective “cured” urinary incontinence showed a significant decrease in the pad test from 22.18 g to 5.55 g (*p* = 0.03, 95% CI 1.54–27.74). All the other patients’ ratings showed an improved pad test.

In Table 3, we further analyze the patients’ ratings and our secondary outcomes. The subjective outcome was more favorable after periurethral bulking than after midurethral polypropylene sling (*p* < 0.001). After bulking therapy, seven patients were cured, four patients showed improvement without the need for further therapy, and one patient required further therapy. With the midurethral sling, only two patients showed an improvement and required no further treatment, three patients noticed no improvement, and four patients worsened after treatment. We also found no correlation between the secondary outcome and the BMI or parity.

After conservative therapy (behavioral therapy, anticholinergic/betamimetic treatment and PFME), nearly 37% of patients felt cured or improved without the need for further therapy, and 68% felt improved, needing further therapy. After bulking, 92% of patients felt cured or improved without the need for further therapy. After the midurethral sling, the outcome was less promising, with 78% of patients feeling the same or even worse.

## 4. Discussion

The findings of our study indicate that 37% of patients diagnosed with CIDO did not require further therapy following conservative treatment, thereby demonstrating the efficacy of this approach as the primary intervention. While bulking agents and midurethral slings have been shown to enhance the outcomes in CIDO, our results suggest that conservative treatment should be the initial step, aligning with the general principles of incontinence therapy. Bulking should be considered the preferred treatment for CIDO. Slings should be used with caution as this was the sole treatment option with increased incontinence following surgery. Nevertheless, this conclusion should be regarded with caution due to the limited sample size and the resulting lack of statistical power.

Epidemiologically, SUI alone is uncommon at an older age as it mostly co-occurs with UUI [1,2,3,4]. Thus, one hypothesis for the relatively high prevalence of MUI is that the stress-induced UUI phenotype is a progressive or advanced stage of MUI, and it is difficult for a woman to distinguish SUI from UUI [2,21]. Nager et al. recommend that a basic office evaluation, without urodynamic testing, constitutes an adequate preoperative workup for women with uncomplicated SUI [22]. Nevertheless, in cases of SUI where mixed symptoms are present, the European Urogynaecological Association (EUGA) recommends a preoperative urodynamic assessment [23]. Urodynamics represents a valuable tool in the evaluation of women with MUI, facilitating the diagnosis of the underlying pathology. Digesu et al. found that detrusor overactivity was present even in a high proportion of women with MUI with stress predominance (29%) and equal severity of symptoms [4]. Urodynamics aids in conducting comprehensive and thorough preoperative counseling and is the best way to anticipate postoperative outcomes [23].

Previous studies investigating pharmacological treatment have proved effective in treating MUI, but not specifically CIDO. Bump et al. evaluated the outcomes of duloxetine among women with stress-dominant MUI. They reported a significant reduction in urinary incontinence episodes compared with the placebo (65% vs. 44%, *p* < 0.05) [21]. Kreder et al. and Khullar et al. demonstrated that tolterodine was effective and well tolerated in treating women with MUI and urge-predominant MUI, respectively [24,25]. Additionally there is evidence of the efficacy of antimuscarinic agents (oxybutynin, solifenacin and feoterodine) in MUI [26]. According to Porena et al., the use of PFME is also strongly recommended for women with MUI, with overall cure/improvement rates ranging from 56 to 70% [26]. Regarding surgical treatment, Rezapour and Ulmsten reported that 85% of patients with MUI after midurethral sling were subjectively completely cured at the 4-year postoperative long-term follow-up [27]. Mohr et al. showed improvement in MUI after bulking therapy according to both subjective and objective outcomes [12].

The strength of this study lies in the prospective recruitment in the specialized urogynecological consultation and the inclusion of all patients with CIDO. This is currently the most extensive prospective study of treatments outcomes in CIDO. The patient-centered approach ensured that each patient’s unique needs and preferences were considered in their treatment plan. Another strength is the simultaneous observation of preoperative and postoperative measurements and subjective parameters concerning CIDO. Because we know that the subjective perception of symptoms often does not correlate with the severity of CIDO, it is essential to pay attention to the patient’s state of mind and relate these observations to the results. A further strength is using standardized state-of-the-art urodynamic measurements.

The principal limitation of our study is the relatively small number of participants. Although this is the largest prospective study on the therapeutic outcome of CIDO, further research, including extensive follow-up studies, is needed. Another limitation of the study is the lack of a validated questionnaire for the secondary outcome measures. The 24 h pad test and micturition diary are reliable tools for assessing the degree of urine leakage and the number of incontinence episodes, respectively [19]. The 1 h pad test can be used because it is quick and standardized. However, there is no strict parallel with the 24 h pad test, and it may underestimate the sphincter weakness later in the day. It may have been beneficial to perform Bonney tests as part of the bladder stress test [28,29,30] in order to enhance the diagnostic process. Nevertheless, as CIDO is not solely indicative of stress incontinence, it is unclear whether the incorporation of this supplementary test would have resulted in a more favorable outcome [31].

The clinical implications of our study are that a significant proportion of patients with CIDO may derive benefit from conservative therapies, including behavioral therapy, anticholinergic/betamimetic treatment and PFME. These treatments have been established for the treatment of UUI and SUI, respectively, and have been proven to be safe and well tolerated. It is therefore recommended that these treatments be used as first-line therapy. Further studies are required to establish the long-term benefits and to determine whether periurethral bulking or midurethral slings could enhance the therapeutic efficacy among patients with CIDO.

## 5. Conclusions

Our study highlights that over one-third of patients with CIDO achieve a cure following conservative treatment. However, the remaining patients with symptoms may benefit from surgical management, including periurethral bulking or a midurethral sling. The findings of this study provide evidence that behavioral therapy, anticholinergic/betamimetic treatment and PFME are the primary recommended treatment options for patients with CIDO, while surgery is a second-line treatment option. Furthermore, the findings provide a robust foundation for advancing our understanding of CIDO and future treatments.

## Figures and Tables

**Table 1:** Patients’ demographics and urodynamic characteristics.

Characteristics	Patients (N = 35)
Age, yrs, median (range)	64 (19–92)
BMI, kg/m^2^, median (range)	26 (19–45)
Nullipara, n (%)	4 (11.43)
Postmenopausal, n (%)	26 (74.29)
Previous surgery	
Endometriosis resection	1 (2.86)
Hysterectomy, n (%)	3 (8.57)
Previous radiation, n (%)	1 (2.86)
Cystomanometric parameters	
Bladder capacity, ml, median (range)	321 (209–510)
Volume at CIDO, ml, median (range)	189 (45–327)
Leakage with CIDO, n (%)	32 (91.43)
MUCP, cmH_2_O, median (range)	39 (8–78)
Pad test before therapy, g, median (range)	23 (2–90)
Pad test after therapy, g, median (range)	2 (0–31)
Therapy	
BT and drugs, n (%)	35 (100)
PFME, n (%)	22 (62.9)
Bulking, n (%)	12 (34.29)
Sling, n (%)	9 (25.71)

Abbreviations: N = number, BMI = body mass index, CIDO = cough-induced detrusor overactivity, MUCP = max. urethral closure pressure, BT and drugs = behavioral therapy and anticholinergic/betamimetic treatment, PFME = pelvic floor muscle training. Missing: 0.

**Table 2:** Primary outcome (pad test).

	Pad Test before Therapy, g, Mean	Pad Test after Therapy, g, Mean	*p*-Value	(95% Cl)
Overall	27.4	5.71	<0.001	14.61–28.77
Therapy:				
BT and drugs	32.43	2.57	<0.001	14.18–45.53
PFME	28.14	6.09	0.38	−25.81–10.18
Bulking	17.42	7.33	0.12	−42.09–5.07
Sling	30.56	4.88	0.8	−14.61–18.74
Patient rating:				
Cured	20.18	5.55	0.03	1.54–27.74
Improved, no further therapy	29.33	5.27	0.27	−7.82–26.68
Improved, needing further therapy	34	9	0.53	−23.04–43.76
Same	34.66	10.33	0.49	−18.6–38
Worse than before	31.25	2.76	0.27	−11.51–39.23

Abbreviations: 95% CI = 95% confidence interval. BT and drugs = behavioral therapy and anticholinergic/betamimetic treatment, PFME = pelvic floor muscle training. Missing: 0.

**Table 3:** Secondary outcome (patients’ rating).

	Patients (n)	Cured (n)	Improved, No Further Therapy (n)	Improved, Needing Further Therapy (n)	Same (n)	Worse Than Before (n)
First line th						
BT and drugs	35	1 (2.86)	6 (17.14)	23 (65.71)	5 (14.29)	0 (0)
Secondary th						
PFME	22	0 (0)	6 (27.27)	16 (72.73)	0 (0)	0 (0)
Bulking	6	6 (100)	0 (0)	0 (0)	0 (0)	0 (0)
Tertiary th						
Bulking	6	1 (16.67)	4 (66.67)	1 (16.67)	0 (0)	0 (0)
Sling	9	0 (0)	2 (22.22)	0 (0)	3 (33.33)	4 (44.44)

Abbreviations: BT and drugs = behavioral therapy and anticholinergic/betamimetic treatment, PFME = pelvic floor muscle training. Bulking = periurethral bulking, Sling = midurethral sling. Missing: 0.

## Data Availability

The raw data supporting the conclusion of this article will be made available by the corresponding author on request, because our data are confidential.
